# Using detrital zircon to reconstruct Neoproterozoic crustal thickness variation in the northwestern margin of the Yangtze Block

**DOI:** 10.1038/s41598-025-98883-3

**Published:** 2025-04-24

**Authors:** Qiang Gu, Fengcun Xing, Xi Wei, Karem Azmy, Kuizhou Li, Aishi Liang, Jiao Wen, Ziqi Liu, Hanxiao Sun, Gesheng Wang, Xinying Liu, Jinchi Yi

**Affiliations:** 1 State Key Laboratory of Oil and Gas Reservoir Geology and Exploitation, Chengdu University of Technology, Chengdu, 610059 China; 2 Institute of Sedimentary Geology, Chengdu University of Technology, Chengdu, 610059 China; 3 Department of Earth Sciences, Memorial University of Newfoundland, St. John’s, NL A1B 3X5 Canada; 4 College of Earth and Planetary Science, Chengdu University of Technology, Chengdu, 610059 China; 5 Sinopec Shengli Oilfield Branch Chunliang Oil Production Plant, Binzhou, 256504 China; 6 Shengli Geological Mud Logging Company of Sinopic Matrix Co., Ltd, Dongying, 257000 China; 7 Harbin Center for Integrated Natural Resources Survey, China Geological Survey, Harbin, 150081 China; 8 Observation and Research Station of Earth Critical Zone in Black Soil, Harbin, Ministry of Natural Resources, Harbin, 150086 China

**Keywords:** Yangtze Block, Neoproterozoic, Detrital zircon, Provenance, Crustal thickness, Geochemistry, Precambrian geology, Structural geology, Tectonics

## Abstract

Trace element geochemistry and chronology of zircon are reliable tools for reconstructing sediment provenance and crustal evolution, particularly in contexts where early crustal and rock records are sparse. We hereby employ in-situ U-Pb dating of Neoproterozoic detrital zircons to refine our understanding of sediment sources, tectonic settings, and crustal evolution along the northwestern margin of the Yangtze Block. Detrital zircons from the Doushantuo and Dengying formations exhibit similar Neoproterozoic age distributions (700–950 Ma). The lithology of the zircon source rocks correlates with the bimodal volcanic rocks extensively developed in the Micangshan–Hannan region along the Yangtze Block’s northwestern margin. Using Eu/Eu* ratios derived from detrital zircons, we reconstructed crustal thickness variations in the northwestern Yangtze Block during the Neoproterozoic. The crustal thickening from 1000 to 850 Ma, thinning between 850 and 730 Ma, and thickening between 730 and 539 Ma. The U/Yb-Nb/Yb, Nb/Hf-Th/U, U/Yb-Hf, and U/Nd ratios of the Neoproterozoic detrital zircons mainly suggest island arc or orogenic features. The zircons younger than 850 Ma suggest progressively depleted mantle-type characteristics and extensional intra-plate. The fluctuated Th/U ratios exhibit a general trend of increase during 1000–730 Ma, which is followed by a decrease. The U/Yb ratios (mostly > 0.1) show a decrease from 1000 to 820 Ma and an insignificant change from 820 to 730 Ma followed by an increase. The estimated crystallization temperatures of the dated detrital zircons, calculated using the Ti-in-zircon geothermometer equation, reveal a general temperature increase during 1000–730 Ma, followed by a gradual decrease. The Ce/Nd ratios (oxygen fugacity) show a fluctuation but a general increase between 820 and 730 Ma possibly due to heat influx from the subduction slab rollback and sediment melting. All the trace element analyses of detrital zircons indicate that subduction along the northwestern margin of the Yangtze Block persisted until 730 Ma and the slab rollback around 850–730 Ma. It also supports the hypothesis that the South China Plate was situated at the periphery, rather than the interior, of the Rodinia supercontinent. After 730 Ma, the Yangtze Block experienced internal extension and rifting, forming rift basins. However, the northwestern margin of the Yangtze Block continued to collide with multiple microcontinents, resulting in crustal thickening. After ca.625 Ma, crust extension led to a decrease in crustal thickness, which is also consistent with the subduction environment indicated by the cumulative distribution function (CDF) plot of detrital zircon ages.

**Supplementary Information:**

The online version contains supplementary material available at 10.1038/s41598-025-98883-3.

## Introduction

The Neoproterozoic was a pivotal period in Earth’s history and biological evolution, marked by significant global events such as the breakup of the supercontinent Rodinia^1–4^, the Snowball Earth phenomenon^5^, and episodes of pulsed oxidation^6^. During this period, the rise of complex macroscopic life occurred, particularly in the Ediacaran, which also witnessed large-scale disturbances in biogeochemical systems^7–9^. The position of the South China Plate within the Rodinia supercontinent remains debated, with two prominent models: the Slab-arc model posits that it was located at the margin of Rodinia^10–23^, whereas the Plume-rift model suggests that the supercontinent’s breakup was driven by a super-mantle plume, positioning the South China Plate at its center^24–30^. The northwestern margin of the Yangtze Block preserves abundant Neoproterozoic rocks, making it an ideal location for studying the tectonic evolution and sedimentary environments during this critical period^11–13^. However, most studies on the crustal evolution of the northwestern margin of the Yangtze Block have primarily focused on magmatic rocks, with limited integration of sedimentary stratigraphy and provenance analysis using detrital zircons to investigate the continuous evolution of crustal thickness.

In the two different models, the northwestern margin of the Yangtze Block shows different crustal evolution patterns, and the evolution process of the tectonic belt in the northern margin is different^4,12,22,31,32^. Since zircon (ZrSiO_4_) is rich in Th and U but commonly low in Pb, so it has very high mineral stability, which is very useful in explaining the earth’s history^33–37^. Zircon U-Pb age and trace elements data provide critical insights into the provenance and crustal evolution of the source region^35,37^. Therefore, evidence for the provenance and crustal evolution of the northwestern margin of the Yangtze Block may be reflected in the geochemistry of detrital zircon^38–41^. The main objectives of current study are (1) to provide zircon U-Pb geochronological evidence for the third member of the Dengying Formation in the Mianyang-Changning intra-cratonic rift; (2) to investigate the provenance and crustal thickness variation of the Northwestern margin of the Yangtze Block using the detrital zircon trace and isotope geochemistry; and (3) to put the evolution of the source terranes into a process-oriented context to ultimately assess the relationship between the evolution of the Neoproterozoic proto-continental crust at the northwestern margin of the Yangtze Block and the assembly and breakup of the Rodinia supercontinent.

## Geological setting

**Fig. 1:**
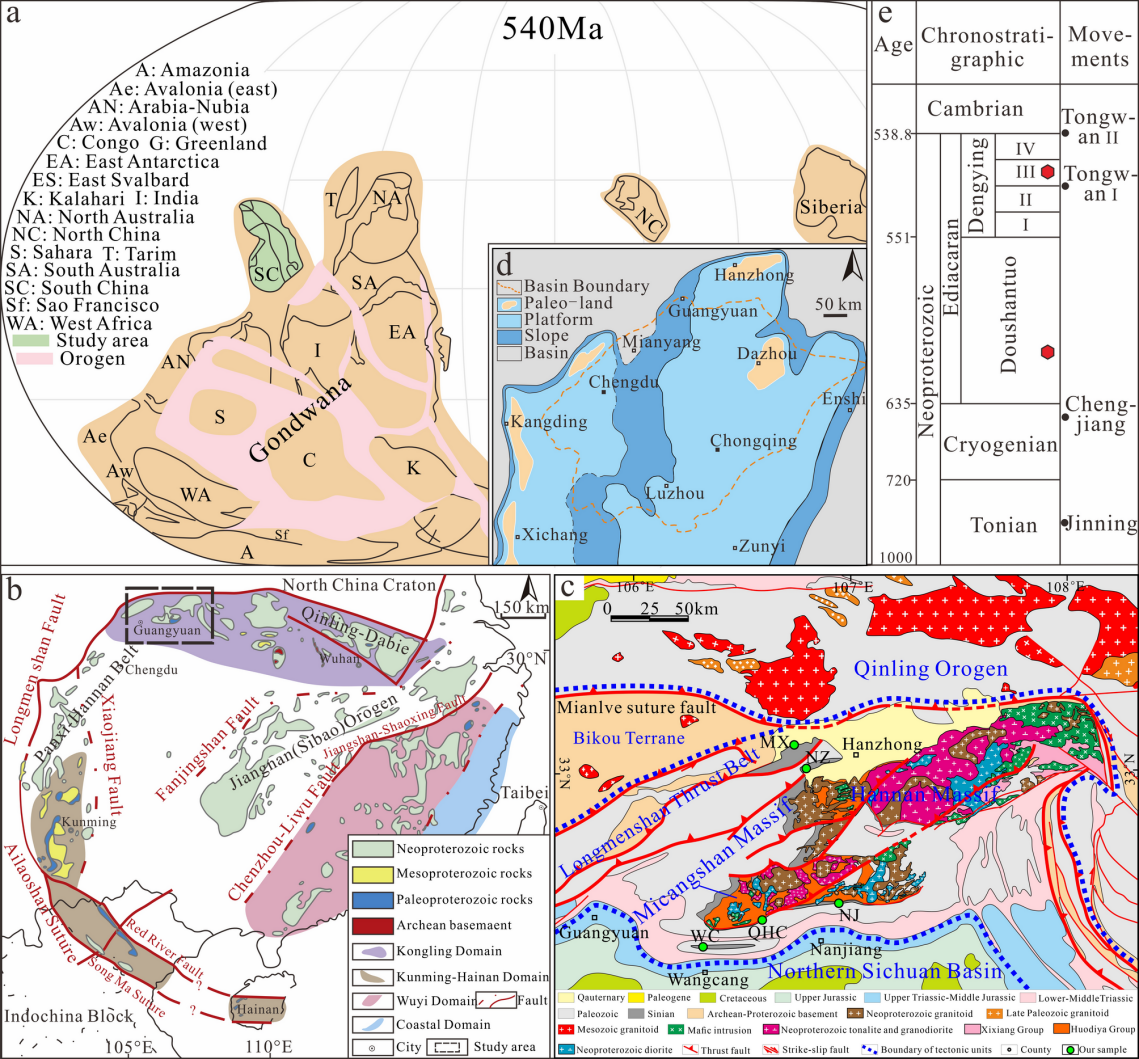
(**a**) Paleogeographic map of Ediacara, the South China Plate is located in the northwest of Gondwana (adapted from Ref^5^. (**b**) Simplified geological map of the South China Plate (adapted from Ref^1^, the study area is located on the northwest margin of the South China Plate. (**c**) Geological map of Hannan area and Micangshan region (adapted from Ref^11,12,24^. (**d**) Sedimentary-tectonic framework of the Sichuan Basin in the late Neoproterozoic (adapted from Ref^47,83^. (**e**) The concluded stratigraphy column with the sample location of the current study, red dots represent sampling locations.

The South China Plate (Fig. 1a) was located in the northwest of Gondwana in the late Neoproterozoic^5,42^. The South China Plate comprises the Yangtze Block in the northwest and the Cathaysia Block in the southeast (Fig. 1b), separated by the Jiangnan Orogen^1,14,43–46^. The study area is located along the northwestern margin of the Yangtze Block within the South China Plate (Figs. 1b–c) and the northern margin of the Mianyang–Changning intra-cratonic rift (Fig. 1d). Beneath the Yangtze Block and its adjoining source regions, Archean to Paleoproterozoic basement rocks is widespread and contribute to the sedimentary units of the block (Fig. 1c), but this basement is only sporadically exposed and does not form an extensive surface area^46^. During the Tonian, the Sibao-Jinning orogeny uplifted South China, forming a landmass with an unconformable contact overlying older stratum. The Jinning orogeny was characterized by significant tectonic activity and widespread magmatism, leading to the intrusion of extensive volcanic bodies. These processes deformed and metamorphosed earlier sediments, producing low-grade metamorphic rocks, higher-grade metamorphic rocks, and migmatites. In the early Cryogenian, the Chengjiang Movement in the Yangtze Block triggered intense volcanic activity, resulting in the deposition of clastic rocks, volcanic tuffs, and lavas^47^. Subsequently, the Nantuo Formation became dominated by clastic glacial deposits. Following the glacial period, a rapid marine transgression during the Ediacaran led to rift basin infill^48^.

The sedimentary environment evolved from clastic deposits of the Doushantuo Formation to carbonate platform deposits of the Dengying Formation (Fig. 1d). This progression established a compensatory carbonate platform under sustained marine transgression and basement subsidence along the northern Yangtze margin, where the Hannan Paleo–land was largely eroded^49–51^. The platform, primarily composed of the Dengying Formation dolomite, shows significant sedimentary differentiation. This was influenced by inherited paleotopography from the underlying Doushantuo Formation, as well as the Tongwan Movement (Fig. 1e), extensional tectonics, and ongoing transgression^47^. Meanwhile, in the southeastern margin of the Yangtze Block, the Liuchapo/Laobao Formation, dominated by dark siliceous rocks, represents a coeval deep-water slope and basin system with the Dengying Formation^52^. The third member of the Dengying Formation in the Yangtze Block, predominantly consisting of clastic rocks, was deposited after the weathering and erosion associated with the first phase of the Tongwan Movement. It has unconformable contact with the underlying dolomite of the second member of the Dengying Formation^53^. Tuffaceous and argillaceous tuff layers at the base of the third member of the Dengying Formation in the western and southeastern Sichuan Basin suggest volcanic eruptions linked to the first phase of the Tongwan Movement^51,54^.

## Methodology and data

### Zircon U-Pb geochronology and trace element geochemistry

All 12 samples were collected from the Mianxian (MX), Nanzheng (NZ), Nanjiang (NJ), Wangcang (WC) and Qunhuacun (QHC) sections, all of which are located on the northwestern margin of the Yangtze Block (Fig. 1c; The exact locations are provided in Supplementary Table 1). Zircons were separated by conventional heavy liquid and magnetic techniques. Grains were handpicked under a binocular microscope, mounted in epoxy resin, sectioned, and polished to approximately half-thickness at Langfang Yuneng Co., Ltd., Langfang, China. Optical photomicrographs and backscattered electron or cathodoluminescence images were acquired to identify and select the least fractured, inclusion-free grains for analysis^55,56^. Laser ablation sampling was performed with a GeoLas HD excimer laser system at Wuhan Sample Solution Analytical Technology Co., Ltd., Wuhan, China, using a 32 μm spot diameter. U–Pb dating and trace element analyses of 1629 zircon grains were conducted simultaneously using an Agilent 7900 laser inductively coupled plasma mass spectrometer (LA-ICP-MS). Data were corrected for instrumental mass bias and depth-dependent elemental and isotopic fractionation using zircon 91,500 as the external standard^3,24^. Trace element quantification was calibrated with the NIST-610 standard glass^43^. Zircon ages were calculated using ICPMSDataCal^57^, and visual analyses were performed with IsoplotR^58^. Each sample was analyzed with more than 130 zircon grains for U-Pb dating to minimize the influence of variations in zircon age components and their proportions^59–62^. Only zircon ages with concordance greater than 90% were used in the geochronological analysis^59–61^. For zircons younger than 1000 Ma, the lower radiogenic ^207^Pb content may lead to larger analytical errors^63,64^. Therefore, their ^206^Pb/^238^U ratios were used to determine the best age, while for zircons older than 1000 Ma, the ^207^Pb/^206^Pb ratios were preferred^64^.

### Multidimensional scaling

Multidimensional scaling (MDS) plots of detrital zircon U–Pb age data are based on the Kolmogorov–Smirnov or Kuiper tests, with distances between points representing the dissimilarity between samples^62,65,66^. MDS has become a critical tool in detrital zircon geochronology, particularly for distinguishing and comparing multiple samples in large datasets^65^. In the current study, a two-dimensional MDS plot was generated using IsoplotR^58^.

### Zircon crustal thickness proxy

Chondrite-normalized zircon europium anomalies (Eu/Eu*) reflect the fractionation of a melt in the presence of specific phases and can be used to reconstruct trends in crustal thinning and thickening^40,41,67–70^. We applied the equation from Tang et al.^39^: *d*_m_ = (84.2 ± 9.2) × [Eu/Eu^*^] _zircon_ + (24.5 ± 3.3) to calculate crustal thickness from Eu/Eu*, where *d*_m_ represents crustal thickness (depth to Moho). Zircon trace element compositions were filtered based on an LREE-Index > 50 (LREE-I = Dy/Nd + Dy/Sm), which effectively removes the majority of chemically altered zircons, often revealing previously obscured magmatic signals^71^. Besides, whole rock La/Yb can distinguish deep from shallow fractionation processes due to differences in the partition coefficient between residual phases and intermediate melt^72^, but zircon Eu/Eu^*^ does not correlate with whole rock La/Yb in S-type granitoids (P contents > 750 ppm)^39,41^, as the S-type granitoids do not necessarily fractionate near the base of the crust and therefore do not record crustal thickness^67^. High La concentrations (> 1 ppm) may be compromised by inclusions, and zircons with Th/U < 0.1 may be affected by metamorphic overgrowth^39,73,74^. Therefore, we also excluded zircons with P contents > 750 ppm, La > 1 ppm, and Th/U < 0.1 from the detrital zircon database before applying the Eu/Eu^*^ proxy^39^.

## Results

### Petrography

**Fig. 2:**
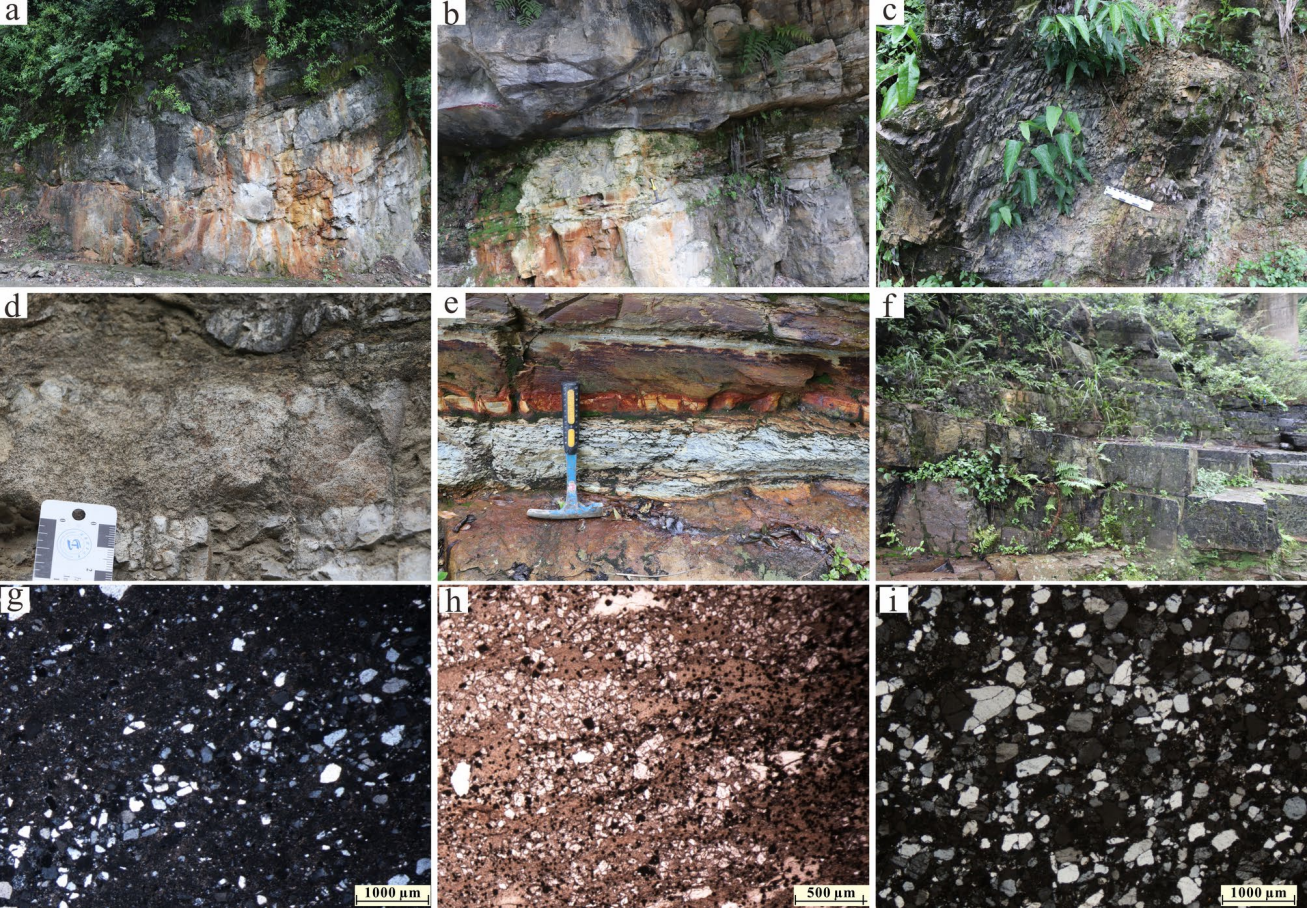
Photographs and photomicrographs of studied samples. (**a**) Thick-bedded quartz sandstone of the Dou Shantou Formation (NZ-Z-3). (**b**) Yellow-gray shale (NZ-Z-7). (**c**) Blue-gray shale (MX-Z-1). (**d**) Sandstone (WC-Z-7). (**e**) Sandstone (QHC-4). (**f**) Layered chert lying on sandstone. (**g**) Sandstone (QHC-1). (**h**) Sandy dolomite containing abundant pyrite (QHC-Z-3). (**i**) Sandstone (NJ-Z-2).

To investigate provenance variations during the Ediacaran on the northwestern margin of the Yangtze Block, we selected a sample from the Doushantuo Formation in the NZ section, along with several additional samples from the Dengying Formation (Fig. 1c). The NZ section sample is composed of thick-bedded quartz sandstone (Fig. 2a). Samples from the Dengying Formation in the NZ and MX sections include yellow-gray shale (Fig. 2b) and blue-gray shale (Fig. 2c), respectively. The WC section primarily consists of sandstone deposits (Fig. 2d). At the base of the clastic rock in the QHC section, the gravel layer is unconformably overlain by algae-bearing dolomite (Fig. 2e). The lower section contains several sandstone layers (Fig. 2g and i) and interbedded sandy dolomite (Fig. 2h), with abundant pyrite grains in the sandstone (Fig. 2h). Overlying this is a layer of laminated black siliceous rock deposits (Fig. 2f).

### Detrital Zircon U–Pb geochronology and trace elements

**Fig. 3:**
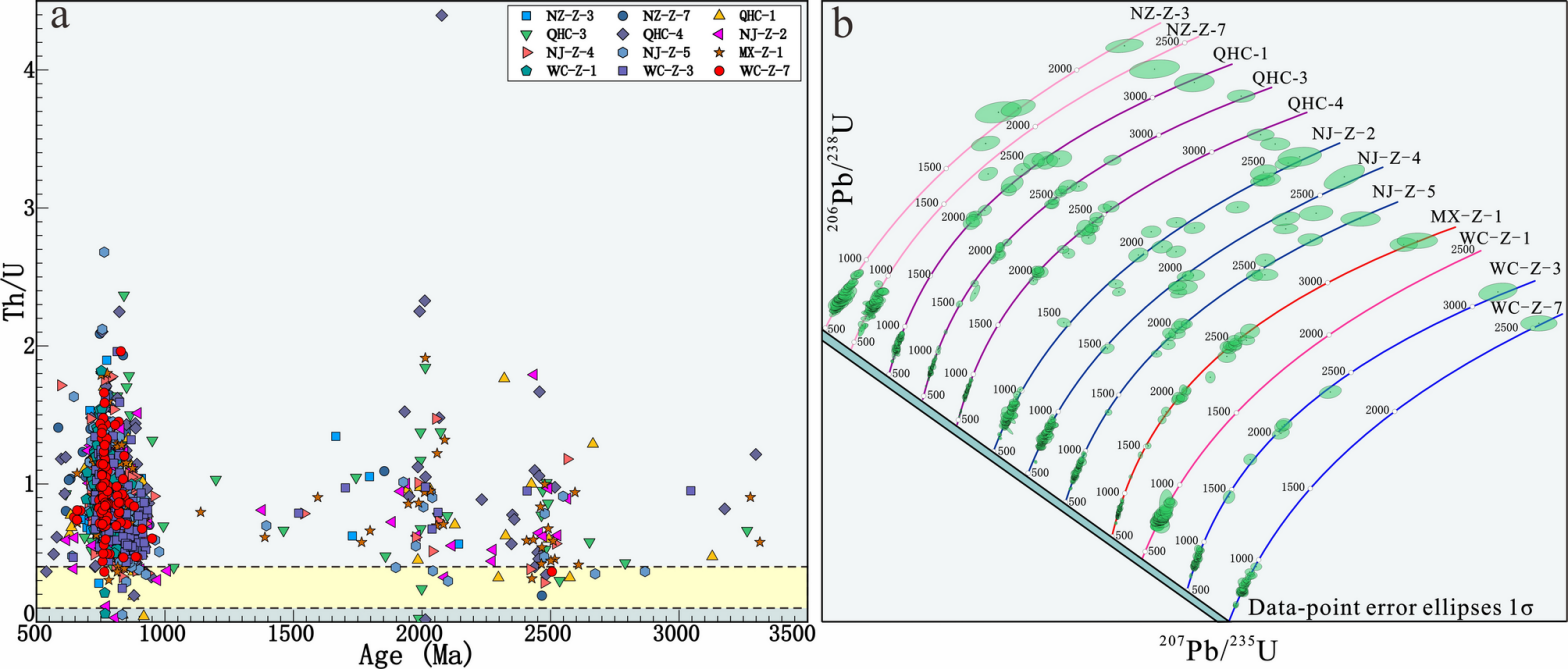
(**a**) Th/U ratio diagram of detrital zircon analyses. (**b**) Concordia plots of samples from the northwestern margin of Yangtze Block. Sample NZ-Z-3 was collected from the Doushantuo Formation, and all other samples were collected from the Dengying Formation.

**Fig. 4:**
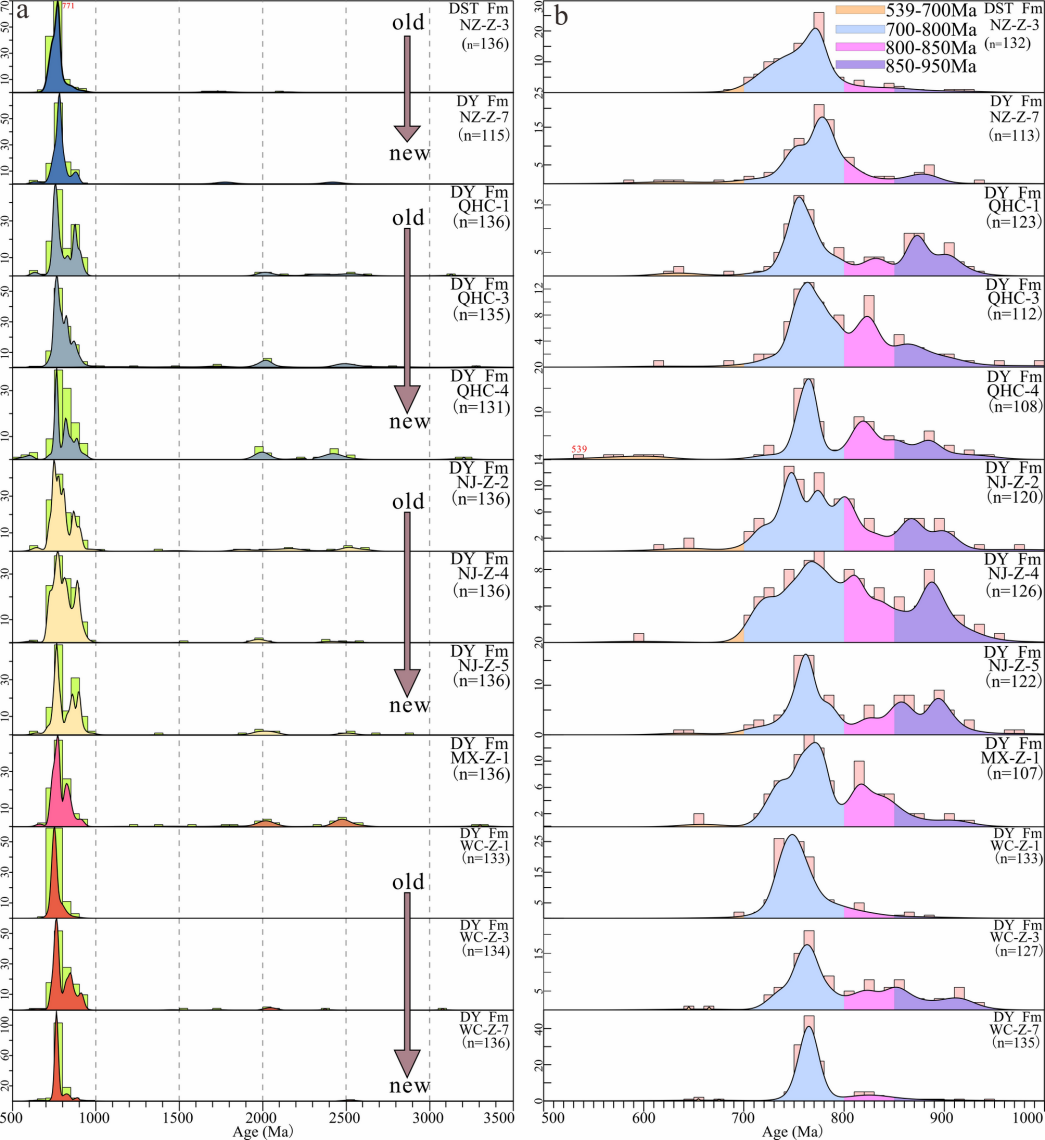
(**a**) Kernel density estimate (KDE) diagrams of detrital zircon U–Pb ages. (**b**) The KDE diagrams of Neoproterozoic detrital zircons corresponding to the left figure.

Most zircons in this study exhibit oscillatory zoning (Supplementary Fig. 1) and high Th/U ratios (> 0.4; Fig. 3a), indicating a magmatic origin^43,74^. Detrital zircons of 12 Neoproterozoic samples from the northern Yangtze Block show consistent age patterns (Figs. 3b and 4). These zircons display a broad range of ages, with the primary source rocks from the Neoproterozoic (538–950 Ma), and minor contributions from the Paleo-Proterozoic (1700–2150 Ma), early Paleo-Proterozoic to late Archean (2300–2600 Ma), and Mid-Archean (3050–3300 Ma) periods (Figs. 4a‒b). Strongly discordant grains (discordance > 10%) have been excluded from the analysis (Figs. 3b and 4). The full analytical data, along with observations of the external morphology and internal structure of each grain, are provided in Supplementary Tables 1 and Supplementary Fig. 1.

The trace-element analysis was conducted concurrently with the U–Pb analysis. All analyzed zircons display similar trace element compositions among the valid data (Supplementary Table 2). The zircon grains are enriched in heavy rare earth elements (HREE), with positive Ce and negative Eu anomalies, which are typical of magmatic zircons^36,75,76^.

## Discussion

### Depositional setting and ages

The study area is located at the northern margin of the Mianyang–Changning intra-cratonic rift in the Yangtze Block (Fig. 1d), and the formation time and mode of the rift are controversial^47,48^. Determining whether the third member of the Dengying Formation was deposited within the rift is essential for understanding the tectonic properties and time of the rift^77^. The tuffs produced by these volcanic eruptions were synchronous and widespread, serving as important markers for stratigraphic correlation. In the northwest margin of the Yangtze Block, although no typical tuff has been found in the clastic rock section of the Dengying Formation, detrital zircon U-Pb ages provide a constraint to the depositional age^78^. The youngest zircon from the QHC-4 sample in the middle of the clastic rock section of the Dengying Formation in the QHC section yields a concordant age of 538.6 ± 5.3 Ma (99%). This is consistent with the age (539.6 ± 1.4 Ma) obtained by Zi et al.^51^ from the volcanic tuff at the base of the third member of the Dengying Formation in the Ebian Pioneer section at the northwest margin of the Yangtze Block and also with zircon ages (542.6 Ma and 542.1 Ma) from tuff in the Liuchapo Formation in the Bahuang and Ganziping sections of Guizhou and Hunan provinces^79^.

**Fig. 5:**
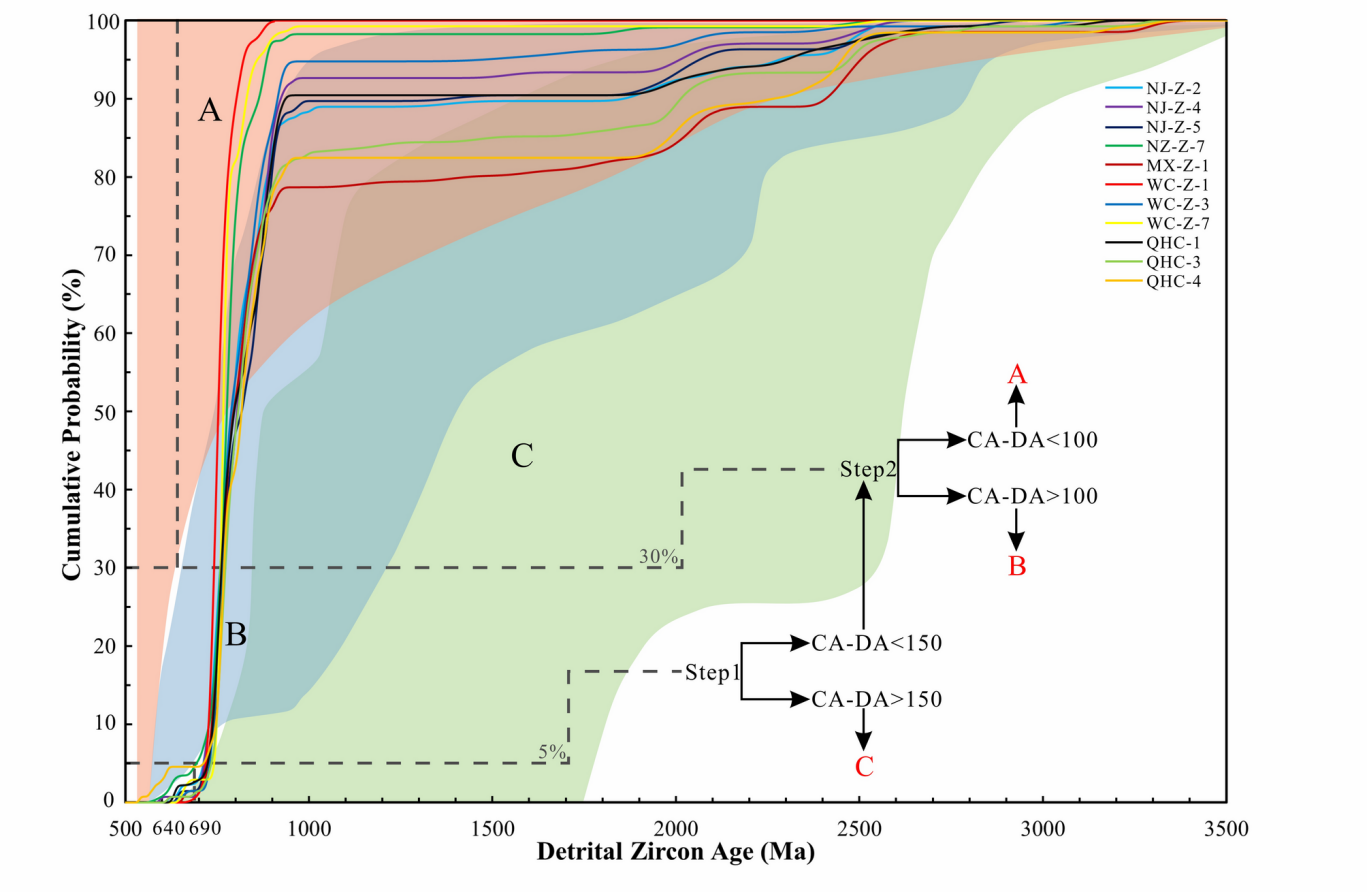
Cumulative distribution plots for each sedimentary in the Neoproterozoic, convergent setting (A: red field), collisional (B: blue field), extensional settings (C: green field) (Basemap from Ref^81^.

Using the cumulative distribution function (CDF) plot of detrital zircon ages, we constrained the tectonic setting in which the sediment was deposited^80,81^. The youngest 5% of detrital zircon crystallization ages from all Dengying Formation zircon samples differ from the stratigraphic depositional ages by more than 150 Ma, indicating that the deposition of the third member of the Dengying Formation at the northwest margin of the Yangtze Block occurred in an extensional tectonic setting (Fig. 5). The deposition of the third member of the Dengying Formation occurred within the Mianyang–Changning intra-cratonic rift with the development of extensional faulting and deeper water environment (Fig. 2f). The undercompensated depositional setting resulted in a sedimentary thickness much thinner than that on both sides of the rift^47,49,50,82,83^. Similar depositional features were reported in the Dengying Formation at the Dashuizha section in the central part of the rift^84^. Therefore, the thickness and spatial distribution of the strata, along with the absence of major tectonic events and igneous activity, suggest that the Ediacaran strata were deposited in an extensional tectonic setting^82,83,85^.

### Zircon source rocks and tectonic settings

**Fig. 6:**
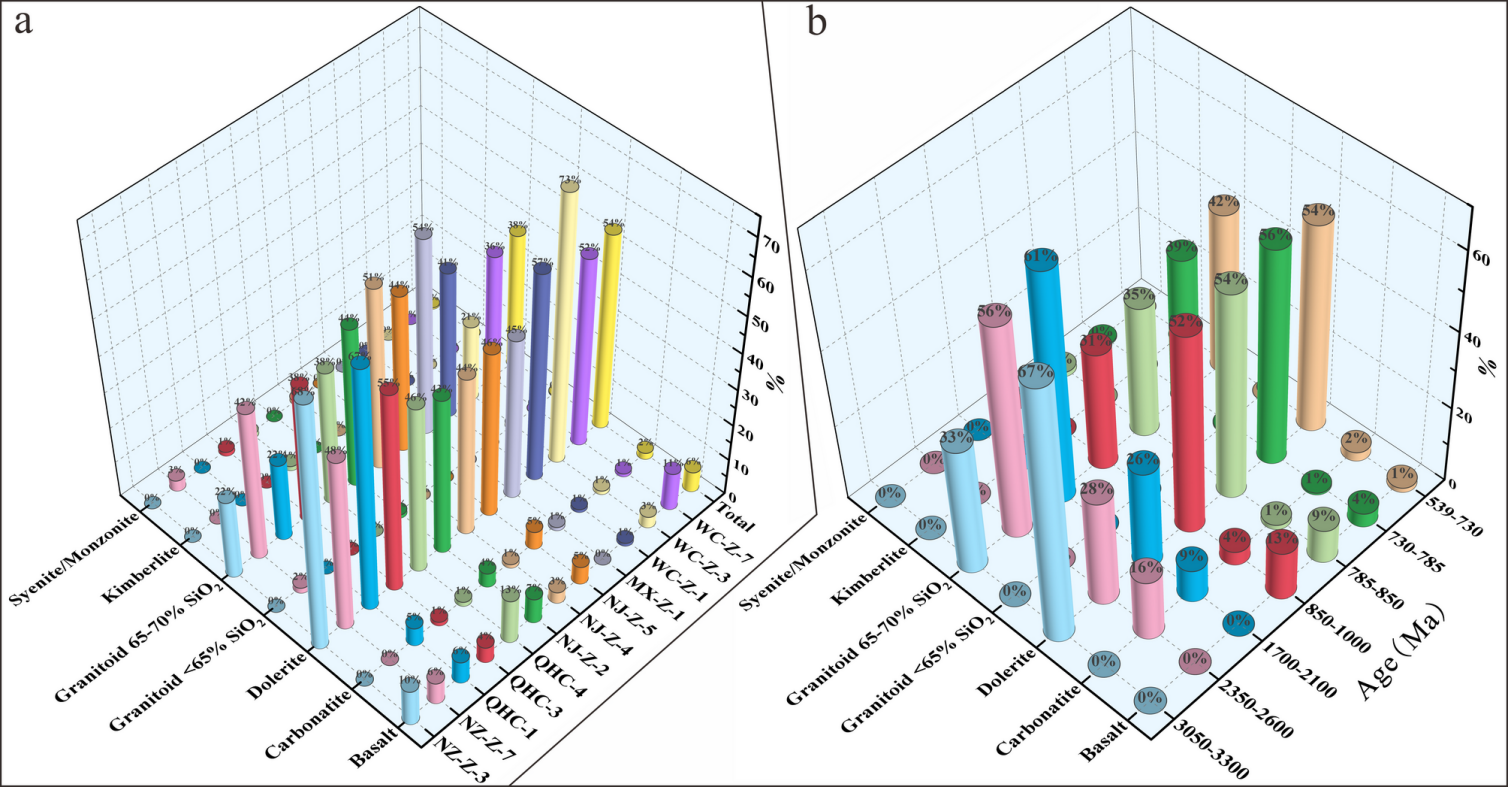
Relative abundance of rock types in different samples according to zircon compositions. (**a**, **b**) Relative abundance of rock types of different ages according to zircon compositions.

**Fig. 7:**
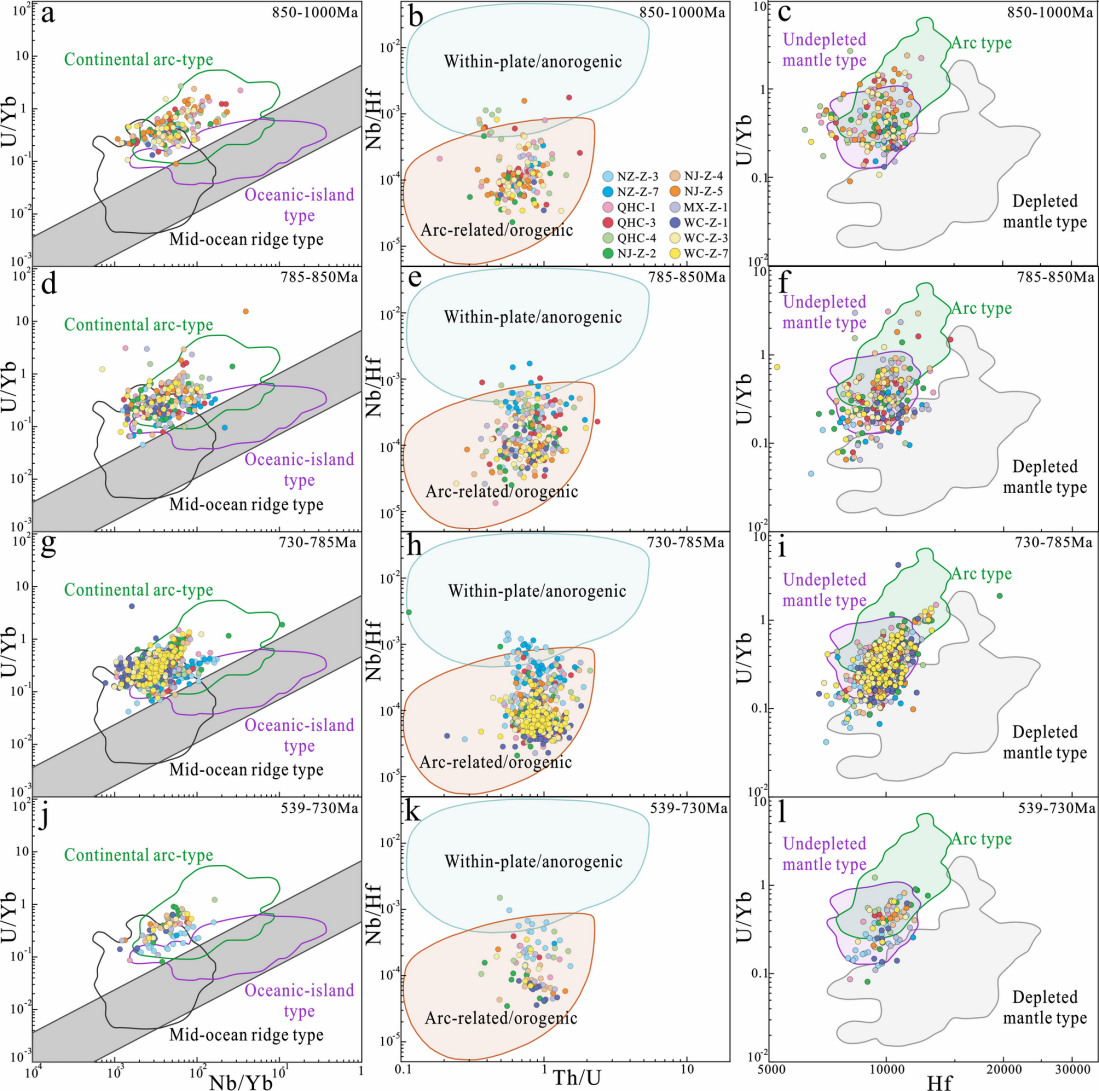
Trace element diagrams of zircon grains of different age zones in the Neoproterozoic. (**a**) U/Yb–Nb/Yb diagram from 850–1000 Ma (Basemap from Ref^97^. (**b**) Nb/Hf–Th/U diagram from 850–1000 Ma (Basemap from Ref^98^. (**c**) Hf–U/Yb diagram from 850–1000 Ma (Basemap from Ref^97^. (**d**) U/Yb–Nb/Yb diagram from 785–850 Ma. (**e**) Nb/Hf–Th/U diagram from 785–850 Ma. (**f**) Hf–U/Yb diagram from 785–850 Ma. (**g**) U/Yb–Nb/Yb diagram from 730–785 Ma. (**h**) Nb/Hf–Th/U diagram from 730–785 Ma. (**i**) Hf–U/Yb diagram from 730–785 Ma. (**j**) U/Yb–Nb/Yb diagram from 539–730 Ma. (**k**) Nb/Hf–Th/U diagram from 539–730 Ma. (**l**) Hf–U/Yb diagram from 539–730 Ma.

Detrital zircon trace element contents broadly constrain the tectonic setting of the magmas from which the zircons crystallized, and the source lithologies. Rare earth element (REE) abundances are influenced by factors such as magma source depth, protolith type, oxygen fugacity, and magmatic water content in the parental melts^33,68,69,74,86–91^. The abundance and ratios of these trace elements are potentially valuable for distinguishing zircons from different sources, thus contributing to studies on sediment provenance^69,71^. Belousova et al.^86^ observed broad correlations between zircon trace-element patterns and the composition of their magmatic host rocks. We applied classification and regression tree methods to zircon trace element data^86^ and found that the majority of zircons from the Doushantuo and Dengying formations originated from granitoid and mafic rocks (dolerite + basalt), indicating bimodal magmatic sources in the region (Fig. 6a). A small number of Early Paleoproterozoic to Late Archean detrital zircons, sourced from carbonate (Fig. 6b), likely originated from the Kongling complex^92^. Li et al.^93^ identified metamorphic carbonates with zircon ages of 2001.3 ± 9.5 Ma in the Kongling complex within the Paleoproterozoic continental collision belt, which is consistent with the age (2.9–2.6 Ga) of the regional basement rocks^94–96^. The geochemistry of zircon is effective for fingerprinting tectono-magmatic provenance^68,76,89,97^. Trace element ratios, including U, Th, Nb, Sc, Ce, and Yb, distinguish zircons from different modern tectono-magmatic settings^76,88,89,97,98^. Correlations of U/Yb-Nb/Yb (Figs. 7a, d, g, j), Nb/Hf-Th/U (Figs. 7b, e, h, d, k), and U/Yb-Hf (Figs. 7c, f, i, l) may provide clues on the nature of the source magma from which they crystallized. Zircons older than the Neoproterozoic typically exhibit compressional characteristics of continental island arcs or orogenic environments, primarily of undepleted mantle type (Supplementary Fig. 2). Neoproterozoic detrital zircons mainly show island arc or orogenic features. However, zircons younger than 850 Ma show an increasing presence of oceanic island arc and depleted mantle-type characteristics. Additionally, extensional intra-plate/non-orogenic detrital zircons appear and increase over time. This trend is closely related to the subduction, compression, and extension of the Yangtze Block’s northwestern margin during the rifting of the Rodinia supercontinent and the assembly of Gondwana.

### Provenance of the Dengying formation

**Fig. 8:**
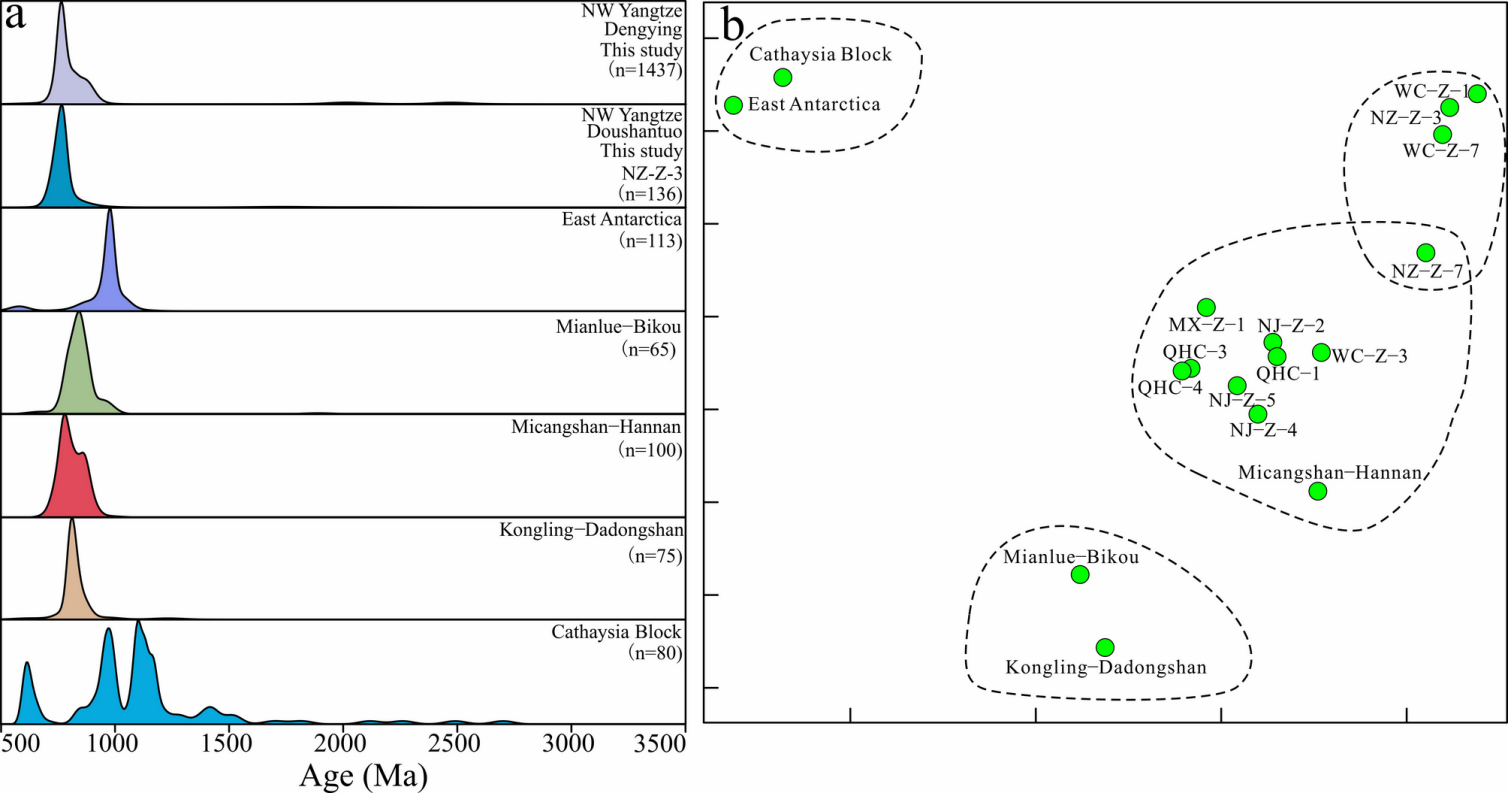
(**a**) The U-Pb age spectrum of detrital zircons of the Ediacaran in the northwestern margin of the Yangtze River (this study), compared with the age probability curves of East Antarctica, Mianlue-Bikou^4^, Micangshan-Hannan^4^, Kongling-Dadongshan^4^ and Cathaysia Block^2^. (**b**) Multidimensional scaling diagram of the sample.

It has been established that the Neoproterozoic sediments of the Yangtze Block originated from an internal source area^2,99^. There were two major source regions (Fig. 1d): the southern source region, which included the Kangdian Paleo-land, Niushoushan Paleo-land, Luding volcanic island arc, and the northern source region that was the Hannan Paleo-land^49^. The study area is closer to the Hannan Paleo-land source region (Figs. 1c‒d). During the deposition of the third member of the Dengying Formation, the Hannan Paleo-land was uplifted, providing a large amount of continental detrital sediment. The high quartz content, large grain size^100^, and high proportion of euhedral grains suggest that the source region was relatively proximal (Fig. 2g and i). Detrital zircon is a powerful tool for understanding provenance and sedimentary dispersal systems^55,63,68,101^. By comparing the U-Pb geochronology data of detrital zircons from the basin and potential source areas, the main contributions to the source-sink system can be identified^102–104^. The detrital zircon ages of our samples indicate that Neoproterozoic magmatic activity was intense in the provenance region (Figs. 4a‒b). These extensive Neoproterozoic rocks, found in the Panxi–Hannan Belt along the western and northern margins of the Yangtze Block (Figs. 1b‒c), were mostly emplaced between 700 and 890 Ma^10–12,28,56,105–111^. In the Micangshan–Hannan areas (Fig. 1c), extensive outcrops of Neoproterozoic acidic granites and basic gabbros (bimodal magmatic sources) are highly consistent with the inferred source rock lithologies based on trace element data. In addition, the Archean crystalline basement is widely present in the Yangtze area (Fig. 1b), but it is less exposed^112^. A small number of detrital zircons aged 3050–3300 Ma and 2350–2600 Ma may originate from island-arc granites and carbonate-rock assemblages (Figs. 1b and 4a) found in the Kongling Complex^113^. Detrital zircons from 1700 to 2100 Ma are derived from the Meso-Neoproterozoic Houhe Complex in the Micangshan–Hannan massif (Figs. 1b‒c and 4a). In the northern Yangtze, widespread magmatic-metamorphic events related to subduction and collision around ca. 2.5 Ga^112,114–116^, and ca. 2.15–1.9 Ga granitic magmatism^95,112,113,117^ have been recorded. The 1.85 Ga bimodal magmatic rocks may represent the completion of the cratonization process in northern Yangtze^43,118^. Based on zircon chronology (Figs. 8a‒b), the nearby Micangshan–Hannan massif likely accounts for the contribution of zircons^10^, which is also supported by the zircon εHf(t) evidence^119^. However, due to variations in the distribution of source rocks, the primary Neoproterozoic detrital zircons exhibit some differences, with certain samples lacking detrital zircon ages older than 800 Ma. In summary, these evidences confirm that the Micangshan–Hannan block is the source region.

### Correlation between crustal thickness variations and rodinia supercontinent cycles

**Fig. 9:**
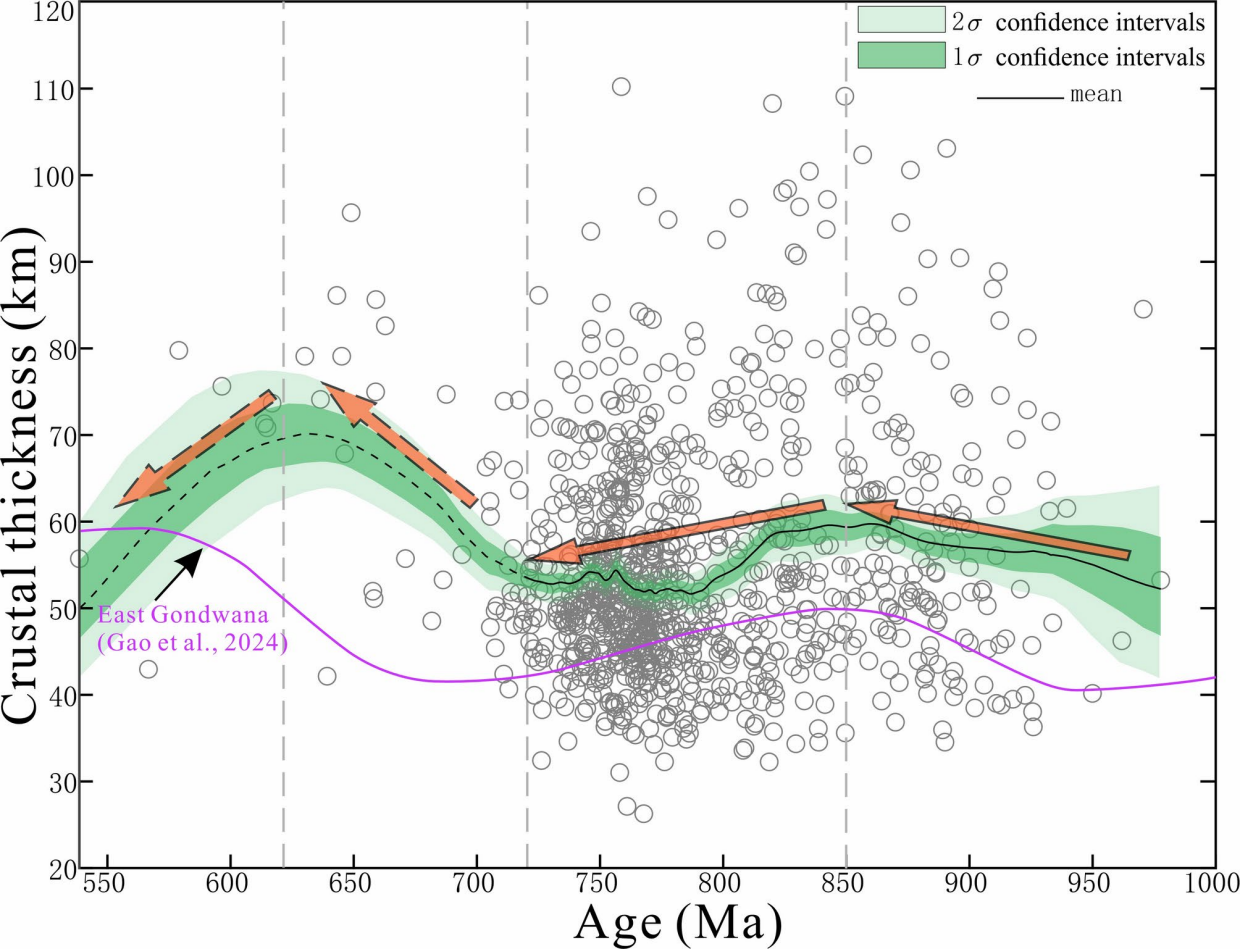
The crust thickness variation of the northwestern margin of the Yangtze Block in the Neoproterozoic and the crust thickness variation of the East Gondwana adapted from Gao et al.^121^. The bootstrapped moving averages of all curves were generated using Acycle software^120^.

Crustal thickness may reflect changes in the driving forces behind the continental evolution of early Earth^38–41^. Long-term variations in crustal thickness are likely linked to prolonged tectonic-magmatic processes, providing key insights into the Neoproterozoic tectonic evolution of the northwestern Yangtze Block. The results from 1,085 data points indicate that the crust thickened rapidly during 950–850 Ma, thinned during 850–730 Ma, and re-thickening during 730–625 Ma (Fig. 9). This trend aligns broadly with the crustal thickness evolution reported for nearby regions^121^.

The rapid crustal thickening around 800–950 Ma (Fig. 9) is interpreted as a response of the northwestern Yangtze Block to the aggregation of the Rodinia supercontinent^28,122–127^. Following this, the breakup of the Rodinia supercontinent influenced a rapid crustal thinning during 820–785 Ma (Fig. 9), with the Yangtze Block undergoing significant extension^18,19,24,30,85,128^. Our crustal thickness data accurately record this thinning (Fig. 9), corresponding to the ca. 820 Ma rift-related magmatism in the northwestern margin of the Yangtze Block^129^, which was coeval with the ca. 817 Ma regional amphibolite-facies metamorphic events and clockwise P-T paths observed in the Micangshan areas^130,131^. These metamorphic events have been linked to regional rifting associated with the continental back-arc rift^92^ and/or extensional orogenic collapse. The subduction system along the northwestern and western margins of the Yangtze Block (Fig. 9) may have persisted until ca. 750–730 Ma^4,11,12,23^.

After 730 Ma, crustal thickening resulted likely from the collision of the northwestern margin of the Yangtze Block with several microterranes, as evidenced by the formation of adakitic (TTG) rocks^12,92^. However, due to the scarcity of detrital zircons younger than 730 Ma, we consider that the crustal thickness inferred from the Eu/Eu* values of our detrital zircons data in this phase is less reliable and should be treated with caution.

The timing of subduction cessation along the northwestern margin of the Yangtze Block varies across different magmatic activity models. Previous studies of volcanic rocks suggest that subduction in the northwestern margin of the Yangtze Block during the Neoproterozoic might have lasted until 750 Ma^12^ or 730 Ma^4^. In the Micangshan region, island arc subduction occurred during 870–820 Ma, gradually transitioning northward to an arc-back arc extensional setting during 840–820 Ma in Xixiang and 825–706 Ma in Hannan^11,12,28^.

**Fig. 10:**
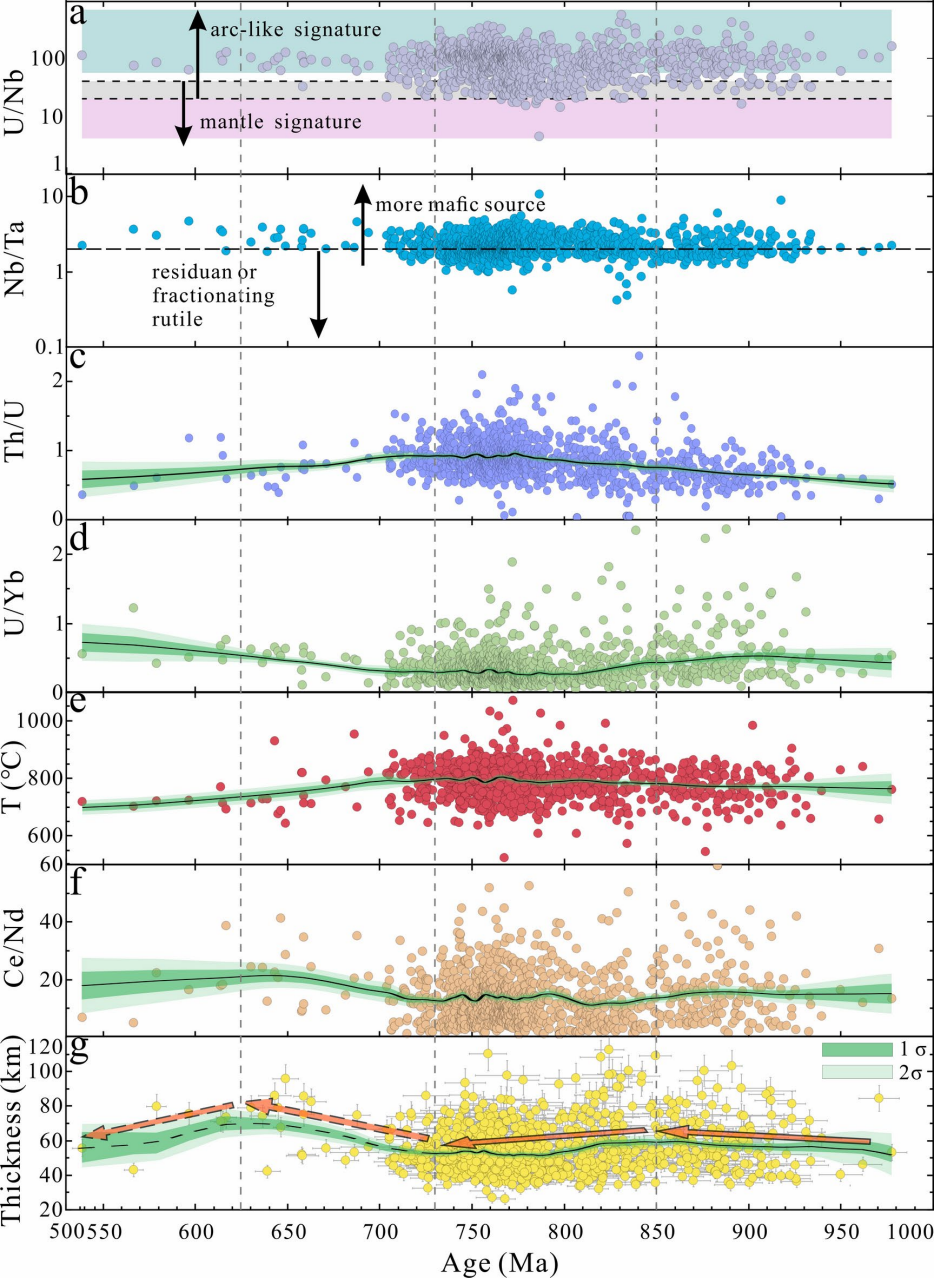
(**a**) U/Nb of detrital zircons. Tectono-magmatic fields are from Grimes et al.^97^. The dashed lines reflect proposed bounding lines between different tectono-magmatic domains. (**b**) Nb/Ta is a melting depth indicator (Basemap from Ref^97^. (**c**) Th/U ratios of detrital zircons. (**d**) U/Yb ratios showing enrichment and/or crustal contamination of the magma from which the zircons crystallized (Basemap from Ref^97^. (**e**) crystallization temperatures of zircons, the equation for the Ti-in-zircon geothermometer is cited from Ref^135^. (**f**) Ce/Nd ratios showing oxygen fugacity of the magma during zircon crystallization^136^. (**g**) Crustal thickness variations. The bootstrapped moving averages of all curves were generated using Acycle software^120^.

In the current work, nearly all Neoproterozoic detrital zircons show U/Nd ratios that are arc-like (Fig. 10a), corresponding to thicker continental crust^39^. Some Nb/Ta values lie below the boundary (Fig. 10b), indicating a magmatic source from mantle-derived mafic melts, consistent with the abundance of mantle-derived mafic volcanic rocks in the Micangshan-Hannan source region^88^. Elevated Th/U ratios typically suggest the incorporation of an older lithospheric component (Fig. 10c), potentially sourced from continental sediments on the subducted slab and/or from the overriding plate^14,131–134^. The Th/U ratios fluctuated and increased during 1000–730 Ma, then gradually decreased (Fig. 10c). We interpret this as a reflection of the transition from a subduction environment/subduction slab rollback to an extensional environment related to rifting along the northwestern margin of the Yangtze Block. This interpretation is supported by volcanic and intrusive rocks (mostly granitoids) in the Micangshan-Hannan massifs, which indicate seemingly uninterrupted magmatic activity from approximately 0.96 to 0.72 Ga^4^. The widespread calc-alkaline I-type granitoids and contemporaneous arc-like mafic rocks in the northwestern domains demonstrate that the tectonic regime was still controlled by a continuous subduction process until the end of arc magmatism at ca. 0.73 Ga^4,11,12^. However, detrital zircons of 730–785 Ma show increasing affinities to both intraplate and arc geochemistry (Figs. 7g, h, i), suggesting that during this period, the Yangtze Block was in a continental rift environment along its northwest margin, influenced by ongoing subduction. This is supported by the low δ^18^O values in volcanic rocks in the northern margin of the Yangtze Block during the same period^4^.

**Fig. 11:**
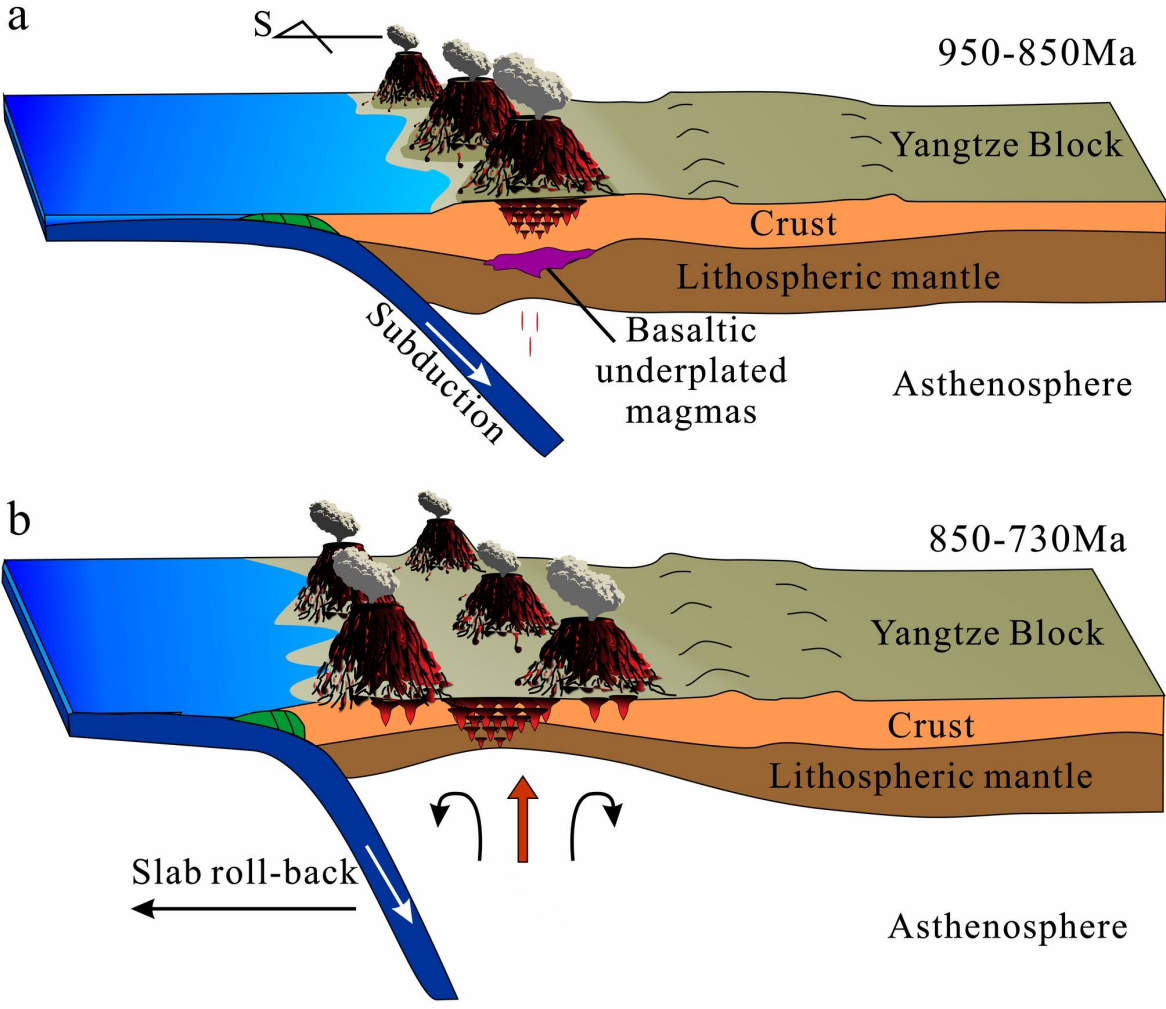
Schematic, not-to-scale cartoon of the (**a**) Ca. 950 − 850 Ma and (**b**) Ca. 850 − 730 Ma evolution of the northwestern margin of the Yangtze Block. Figure created by CorelDRAW Graphics Suite Software 2025 version, URL: https://www.corel.com/akdlm/6763/downloads/free/trials/GraphicsSuite/2025/LJu8$4y2/wt2dt/CDGS2025.exe.

Elevated U/Yb (> 0.1) ratios suggest derivation from enriched or crustal-contaminated magma sources^97^. The currently investigated zircons show that their U/Yb ratios (mostly > 0.1) decreased from 1000 to 730 Ma but increased after (Fig. 10d). The estimated crystallization temperatures of the dated detrital zircons, calculated using the Ti-in-zircon geothermometer Eq. 1^36^, reveal a general temperature increase during 1000–730 Ma, followed by a gradual decrease (Fig. 10e). The Ce/Nd ratios positively correlate with oxygen fugacity^136^. The fluctuating increase in oxygen fugacity between 850 and 730 Ma may be due to heat influx from the subduction slab rollback and sediment melting (Fig. 10f). Thus, the trace element data support the idea that the northwestern Yangtze Block underwent continuous subduction during 1000–730 Ma (Figs. 11a–b), with subduction slab rollback during 850–730 Ma (Figs. 10g and 11b).

After 730 Ma, the Yangtze Block began to undergo internal extension and rifting, forming a rift basin, and gradually evolved into a volcanic rifted margin^4,99,137^. Rifting within the arc may have occurred during 686–630 Ma^31,32^. Several weak age signals also indicate global extreme climatic changes^138^. However, the northwestern margin of the Yangtze Block continued to collide with multiple microcontinents^12,92^, resulting in crustal thickening (Figs. 9 and 10g). After ca. 625 Ma, due to the extension, the thickness of the crust decreases (Figs. 9 and 10g). In addition, our evidence supports the slab-arc model, which posits that the South China Plate was positioned at the margin of the Rodinia supercontinent and that the northwestern margin of the Yangtze Block experienced prolonged subduction^13–19^.

## Conclusions

A detailed geochronological and trace element geochemical study has been applied to the Ediacaran sedimentary detrital zircons from the northwestern margin of the Yangtze Block and led to the following key conclusions:

1. The occurrence of detrital rocks in the Dengying Formation of the Wangcang area in the northern margin of the Mianyang–Changning intra-cratonic rift, along with the appearance of layered siliceous rocks, suggests a relatively deep sedimentary environment.
2. The detrital zircon source rocks exhibit bimodal volcanic characteristics. Both zircon age and geochemical proxies support the origin of sediments from the Micangshan–Hannan massif.
3. The Eu/Eu^*^ ratio of the detrital zircons accurately records variations in crustal thickness, which correlate with the Rodinia supercontinent cycle.
4. Subduction along the northwestern margin of the Yangtze Block may have persisted until 730 Ma, with a slab rollback between 850 and 730 Ma causing crustal thinning, followed by stabilization. Crustal thickening during 730–625 Ma might have been associated with microcontinent collisions.
5. The trace element record of the detrital zircons supports the model of locating the South China Plate at the edge of the Rodinia supercontinent.

## Electronic supplementary material

Below is the link to the electronic supplementary material.

Supplementary Material 1

Supplementary Material 2

Supplementary Material 3

## Data Availability

The datasets used and analysed during the current study are available from the corresponding author on reasonable request.
