# Supplementary material for: Using detrital zircon to reconstruct Neoproterozoic crustal thickness variation in the northwestern margin of the Yangtze Block
Source: Sci Rep. 2025 Apr 24;15:14240. doi: 10.1038/s41598-025-98883-3 (PMC12022102; doi:10.1038/s41598-025-98883-3)
Supplement: Supplementary file 1 — Supplementary Material 1 [file 41598_2025_98883_MOESM1_ESM.docx]

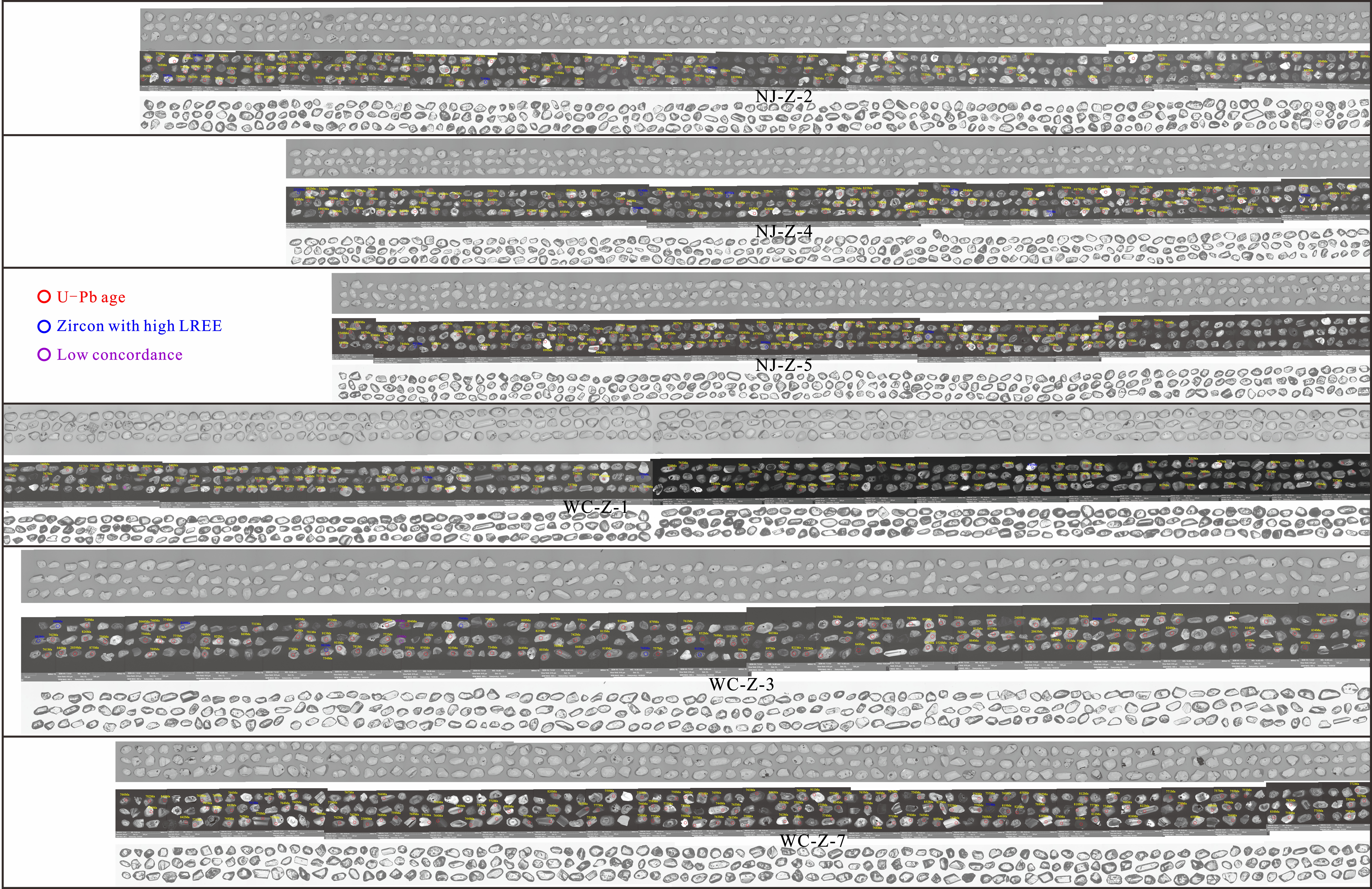

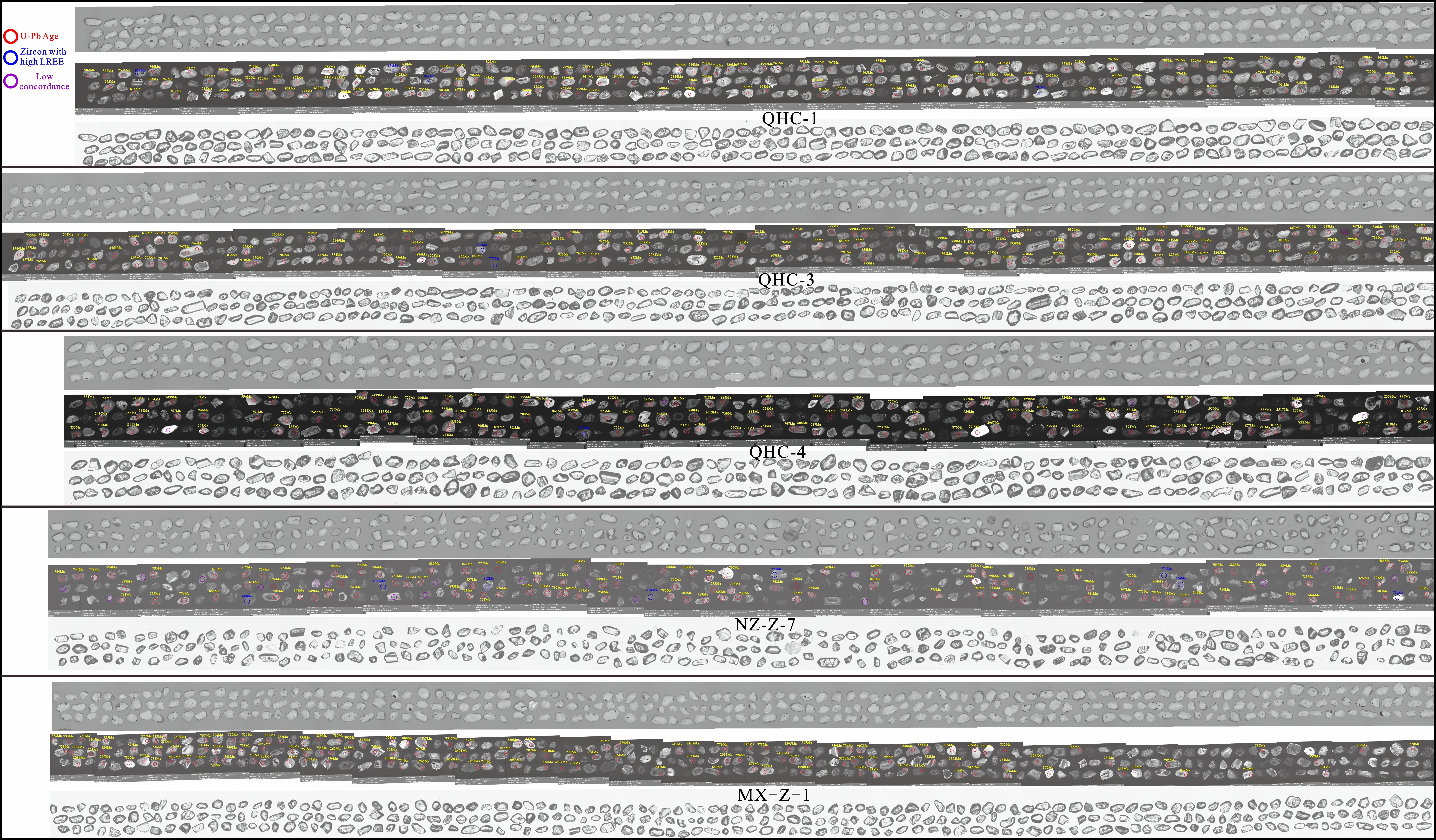


**Fig. S1.** Cathodoluminescence and microscopic images of clastic rock.


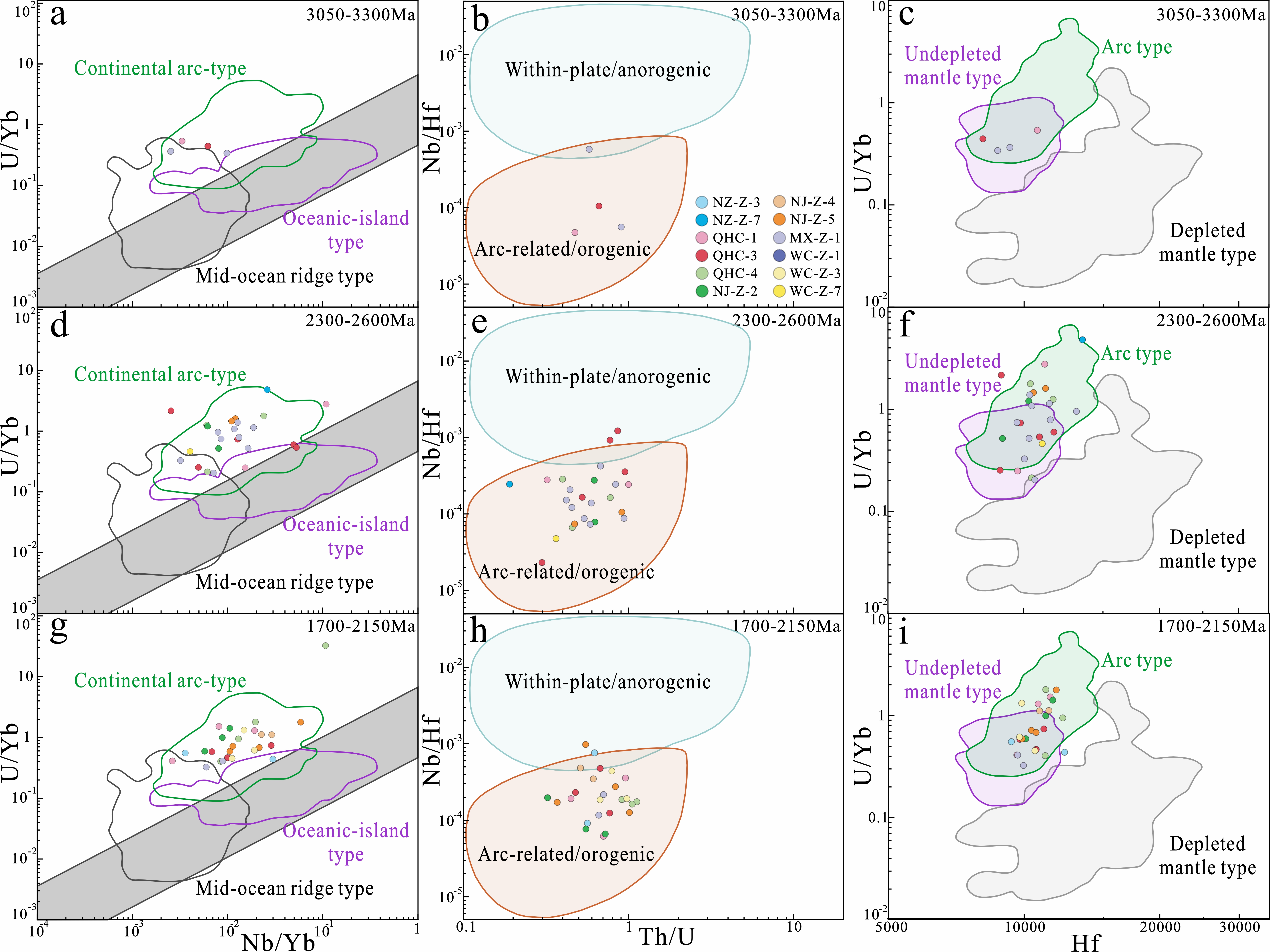


**Fig. S2.** Trace element diagrams of zircon grains of different age zones in the pre-Neoproterozoic.
